# Past, present, and future of microbiome-based therapies

**DOI:** 10.20517/mrr.2023.80

**Published:** 2024-03-18

**Authors:** Pilar Manrique, Ignacio Montero, Marta Fernandez-Gosende, Noelia Martinez, Claudio Hidalgo Cantabrana, David Rios-Covian

**Affiliations:** Department of R&D, Microviable Theapeutics S.L., Gijón 33203, Spain.

**Keywords:** Microbiome-based therapies, microbiota, LBP, MBP, biological drugs

## Abstract

Technological advances in studying the human microbiome in depth have enabled the identification of microbial signatures associated with health and disease. This confirms the crucial role of microbiota in maintaining homeostasis and the host health status. Nowadays, there are several ways to modulate the microbiota composition to effectively improve host health; therefore, the development of therapeutic treatments based on the gut microbiota is experiencing rapid growth. In this review, we summarize the influence of the gut microbiota on the development of infectious disease and cancer, which are two of the main targets of microbiome-based therapies currently being developed. We analyze the two-way interaction between the gut microbiota and traditional drugs in order to emphasize the influence of gut microbial composition on drug effectivity and treatment response. We explore the different strategies currently available for modulating this ecosystem to our benefit, ranging from 1st generation intervention strategies to more complex 2nd generation microbiome-based therapies and their regulatory framework. Lastly, we finish with a quick overview of what we believe is the future of these strategies, that is 3rd generation microbiome-based therapies developed with the use of artificial intelligence (AI) algorithms.

## INTRODUCTION

The exploitation of bacteria by humans is a long-time running business, from fermentation of food products, such as cheese or beer, to heterologous production of compounds with pharmacological means, including antibiotics or insulin^[1-4]^. On top of that, the constant exposure to bacteria - either pathogenic or commensal bacteria that live in our body - constantly affects our daily lives^[5-7]^. In this regard, the scientific community has been extensively studying the gut microbiota-host relationships for several decades, finding strong relationships between bacterial composition and health status^[6-8]^. Particularly, dysbiosis, defined as an imbalance of the microbiota composition and functional capacity, has been related to serious health issues. These include inflammation due to loss of gut homeostasis^[9-11]^, and infections by opportunistic pathogens, such as *Clostridioides difficile* (*C. difficile*), due to loss of protection conferred by the microbiota^[12]^.

Alterations of the gut microbiome that result in the loss of colonization resistance provided by this ecosystem often lead to the acquisition of antibiotic resistance genes by *C. difficile* and subsequent recurrent infections, which is a serious issue that affects 500,000 people every year only in the United States^[13]^. The lack of solutions for this problem has led to the development of new strategies, which include microbiome-based therapies that leverage the naive functionality of a healthy microbiota and are one of the most promising alternatives to date^[14-16]^. The great success of this story has opened the door to a new generation of therapies, which can be designed to treat other types of diseases related to the loss of gut microbiota homeostasis, such as cancer.

In this review, we provide a general overview of how the gut microbiome can be leveraged to treat infectious diseases and reduce the use of antibiotics worldwide. We summarize what is currently known about the influence of the microbiota on the development of certain cancers and how they can alter, for good or for bad, the response to different anticancer treatments. We summarize the past, present, and future of therapies based on gut microbiota and the regulation issues that have arisen with these therapies. Finally, we conclude by outlining the direction we believe the field is heading towards.

## THE ROLE OF THE MICROBIOTA IN INFECTIOUS DISEASES AND CANCER

Even though it is known that the taxonomical profile of the human microbiome is specific to each individual, general composition and functional patterns associated with healthy states have emerged in the human microbiome. Significant alterations in this ecosystem, also known as the dysbiosis state, have been correlated with multiple diseases, and great research efforts are directed towards understanding how to return a dysbiotic microbiome back to equilibrium in order to maintain health^[17]^. The role of the gut microbiome in pathogen protection, also known as “colonization resistance”, has been established for decades and is of most relevance in infections with opportunistic gut pathogens such as *Clostridium difficile* (see 2nd Generation Products section) or urinary tract infections (UTIs) often presented after antibiotic treatment that decimates the gut microbial diversity^[18,19]^. Similar scenarios have been reported with sexually transmitted diseases (STDs) and the vaginal microbiome, in which women with a less diverse vaginal microbiome are generally more susceptible to UTIs and STDs such as human immunodeficiency virus (HIV), herpes simplex virus (HSV), or papillomavirus^[20]^. Treatments that reestablish the complexity of the microbiome in these environments have shown remarkable results in preventing reinfections^[21,22]^.

The great influence of the microbiome in many diseases led to the study of the cancer-gut microbiome axis. In the past decade, data supporting the influence of the gut microbiome on tumor progression and on the response to oncological treatments have been rapidly growing and have been extensively reviewed elsewhere^[23-25]^. In summary, the gut microbiome can have direct and indirect effects on several cancer cells and can modify the activity of different immune cells, ultimately affecting the spread of cancerous cells^[23-25]^. Antibiotic treatment can reduce the effect of immune checkpoint inhibitors (ICI) treatment, such as PD-L1 or CTLA-4 blockade^[26,27]^. Moreover, clinical studies have unraveled differences in the microbiota composition of individuals who respond to treatment compared to those who have not been reported in several studies^[23,28,29]^. Differences in this response can be eliminated with fecal microbial transplantation (FMT) administration. Additionally, oral administration of certain bacterial genera, such as *Bifidobacterium*, *Akkermansia*, and *Bacteroides*, has been shown to improve the efficacy of anti-PDL1 and CTLA-4 treatments^[24,30,31]^. Similar results were reported by Tanoue *et al.*, in which administration of a mix with 11 different strains, belonging to *Alistipes*, *Bacteroides*, *Eubacterium*, *Fusobacterium*, *Parabacteroides*, *Phascolarctobacterium*, and *Ruminococcaceae* groups, enhanced therapeutic efficacy of ICI^[32]^.

## HOST-MICROBIOTA INTERACTIONS WITH THERAPEUTIC TREATMENTS

Even though there is increasing evidence demonstrating that the gut microbiota can affect drug efficacy, the mechanisms by which this happens remain unexplained in many scenarios. Understanding the specific ecological variables and mechanisms by which microbes can influence drug efficiency will aid in the development of successful microbiome-based therapies. Below, we summarize the most relevant microbiome-drug interaction mechanism known, with a special focus on oncological treatments, but we also refer to others when necessary to illustrate a particular known mechanism.

The effect of host microbiota on drugs has been reported since the 80s, together with the discovery of the extensive enzymatic repertoire of the gut microbiota^[33]^. Pharmacogenomics is the way human genetic variation affects drug action and their effectiveness. This idea was the foundation for pharmacomicrobiomics (http://pharmacomicrobiomics.com), or the way variations in gut microbiome composition affect the action and effectiveness of therapeutic drugs^[34,35]^. The modification of therapeutic drugs by the microbiota can result in different types of effects, which is the base of some toxicity effects and the phenomenon known as the “responder-no responder” effect^[35]^.

### Transformation of therapeutic drugs by the gut microbiota

In an extensive study, Zimmermann *et al*. evaluated the ability of 76 diverse human gut bacteria isolates to metabolize 271 orally delivered drugs selected based on their clinical indication, physicochemical properties, and predicted intestinal concentrations. This study shed light on the variability of these interactions and how they affect their efficacy. Specifically, two-thirds of the drugs were partially or completely metabolized by at least one bacterial strain and each bacterial strain was able to metabolize from 11 to 95 drugs^[36]^. The outcome of these metabolizations or interactions can be grouped into three types of effects (increased bioavailability, increased toxicity, or drug inactivation), for which we illustrate some examples below, with a special focus on oncological treatments.

First, the drug could augment its biological activity after metabolization, thus improving its effect. Sulfasalazine, a prodrug used in arthritis, is metabolized by some members of the gut microbiota, improving its bioavailability^[37]^. Metformin is also a case in which microbiota plays a crucial role, but in this case, the relationship is more complex. Metformin changes the composition of the gut microbiota, reducing the abundance of proteobacteria and, simultaneously, changing the profile of short-chain fatty acids (SCFAs) towards more production of butyrate and propionate, and bile acid metabolism resulting in an elevated concentration of total bile acids. These changes are associated with beneficial effects on type-2 diabetes patients, but also with the improvement in other aspects such as cognition and leaky gut^[38-40]^. Regarding oncologic treatments, the chemotherapeutic cyclophosphamide effectivity is boosted by the ability of some bacteria to recruit type 1 T helper (Th1), type 17 T helper (Th17), and CD8+ T cells. These effects have been described in several bacteria, such as *Lactobacillus johnsonii*, *Enterococcus hirae*, and *Barnesiella intestinihominis*, respectively^[41]^. Regarding novel cancer therapies, such as ICI, several recent papers associate the composition of the gut microbiota with the effectivity of these types of treatments^[29,31]^.

On the contrary, some drugs can be inactivated by bacterial metabolization, as is the case of the reduction of digoxin to its inactivated form^[42,43]^. Levodopa, which has to be transformed into dopamine in the brain to treat Parkinson’s disease, can be transformed prematurely by the gut microbiota, herein losing its biological activity^[44,45]^. Gemcitabine, used in pancreatic ductal adenocarcinoma, presents chemoresistance because the long isoform of the enzyme cytidine deaminase is present predominantly in Gammaproteobacteria^[46]^.

Unfortunately, the gut microbiota also interacts with drugs, producing some toxic effects on the host^[47]^. This is the case with the intravenous drug Irinotecan (CTP-11), a drug to treat colorectal cancer. The innocuous metabolic subproducts of CTP-11 (SN-38G) are released to the gut with the bile fluid and are reconverted by the microbiota into their active toxic form (SN-38), causing epithelial damage and diarrhea. An increase in *Veillonella*, *Clostridium*, *Butryicicoccus*, and *Prevotella* species was seen in the CTP-11 group. This effect could be mitigated in a mouse model by modulating the composition of the microbiota with a mix of *Lactobacillus* species, suggesting that modulation of the microbiota with microbiome-based organisms can reduce the side effects caused by this therapy, and the development of such cotreatment should be explored (see sections “1st Generation Products- Interventions to modify gut microbiota” and “2nd Generation Products- Microbiome Based therapies” for more specific ways on how to achieve this)^[48-50]^. Another example consists of the teratogenicity of nitrazepam, which is enhanced by microbial nitroreductases that increase the production of 7-aminonitrazepam, its teratogenic derivative^[51,52]^.

Overall, it has been demonstrated that the gut microbiota composition and the resulting differences in metabolic activity and immune regulation are crucial factors in the therapeutic effects of drugs^[35]^. This effect can be caused by direct metabolization of the drug or indirectly by influencing microbiome-based modulation of the immune system^[38-40]^. Often, it is a two-way interaction between drugs and bacteria, wherein the drug causes changes in gut microbiota composition, and these changes affect the performance of the therapeutic drug^[34,47,53]^. The specific mechanisms by which the microbiota can influence the effect of therapeutic drugs are truly diverse and are summarized in the following section.

### Mechanisms of interaction between therapeutic drugs and gut microbiota

Alexander *et al.*’s review presents the TIMER framework to classify the mechanisms by which the microbiota affects chemotherapy drugs. This classification could be applied to other types of drugs and/or xenobiotic compounds with biological activity. TIMER stands for: Translocation, Immunomodulation, Metabolism, Enzymatic degradation, and Reduced diversity and ecological variation^[53]^. Understanding these mechanisms is essential to define strategies to modulate the microbiota and reduce the toxic effects of drugs, enhance their beneficial effects, and avoid their inactivation which nullifies their effectiveness altogether.

Translocation, the process by which some bacteria cross the intestinal barrier, can be caused by many drugs. Particularly, some chemotherapy drugs, such as cyclophosphamide or doxorubicin, provoke a local inflammation effect on the gut due to the shortening of the villi, with concomitant changes in the microbiota composition^[53]^. Antiretroviral therapy can also cause inflammatory gut barrier damage and, in addition, interfere with gut homeostasis recovery as they also change microbiota composition^[54]^. Opioids are another type of drug that provokes the translocation of bacteria due to the inhibition of myosin light chain kinase (MLCK), a key protein in the maintenance of tight junctions^[55,56]^.

Immunomodulation refers to the effect of certain bacterial groups on immune responses to ensure the effectiveness of chemotherapy. For example, certain bacterial taxa influence the accumulation of Th1 and Th17 cells in the tumor environment^[53]^. In addition to this, understanding immunotherapy cancer drugs becomes intriguing when considering the interplay with microbiota composition. This is because the efficacy of these drugs heavily relies on the activation of specific immune cells driven by certain members of the gut microbiota. As an example, one of the main differences found in responders to immunotherapy of several cancers was their ability to recruit CD8+ cells into the tumor microenvironment^[31,57]^. The authors hypothesized that this recruitment could be mediated by several bacterial populations, but further investigations are needed to establish a robust relationship between bacterial composition and the recruitment of immune cells^[58,59]^. On the other hand, immunomodulation by gut microbiome can result in increased toxicity of chemotherapy and other drugs, leading to conditions such as severe intestinal inflammation that often happens during chemotherapy^[60,61]^.

Bacterial metabolism can be particularly important in the modulation of drug effects. For example, vitamin B from bacterial origin is related to the prevention of colitis during CTLA-4 blockade therapy in melanoma patients^[62]^. Regarding immunomodulation, SCFAs play a crucial role in the efficacy and side effects of several drugs^[40,63,64]^. Acetic, propionic, and butyric acids are the major products of bacteria fermentation in the colon and can result in the modulation of Th1, Th17, and regulatory T cell (Treg) responses^[65]^. This effect has been observed as well for drugs such as metformin or oxaliplatin^[38,39,66]^ and preliminary data suggest that this drug effect might be driven through changes in bacterial metabolites, such as SCFA, but these interactions must be examined in depth. Other examples of microbial metabolites include desaminotyrosine, which acts through the activation of T cells, and desoxythymidine triphosphate (dTTP), produced by microbiota from dietary serine which could be potentially harmful due to the increasing toxicity of chemotherapeutic 5-fluoridine-5’-monophosphate^[67]^. Both examples are explored in the preclinical state.

Bacterial Enzymatic activity, as we commented previously, can transform drugs into active and toxic forms or metabolize active forms to other metabolic subproducts, leading to a loss of efficiency^[34,36]^.

Reduced diversity refers to the loss of several bacterial populations after the administration of drugs, such as in some chemotherapy therapies^[60]^. However, antibiotics are currently the drug that has the most dramatic effect on microbial composition^[68,69]^. These effects include a reduction in SCFA metabolism, an increase in stress response pathways, the emergence of pathogen infections, and the development of antimicrobial resistance^[9-11,70]^. Other drugs, such as proton pump inhibitors, metformin, selective serotonin inhibitors, and laxatives, also change the microbiome composition, leading to a reduced diversity^[34]^.

Unlike host genes, the microbiome can be changed, as seen until now, to achieve a beneficial effect in humans. Thus, the information about drug-microbiota interactions and its mechanisms are key to defining the best strategies to develop microbiome-based therapies as treatments and/or coadjutants of therapeutic drugs and confers some of the basis for personalized medicine^[66,71]^. In the next sections, we provide a summary of the different strategies that have been developed to modulate the gut microbiota for our benefit.

## 1ST GENERATION PRODUCTS - INTERVENTIONS TO MODULATE GUT MICROBIOTA

### Dietary interventions

**Figure 1:**
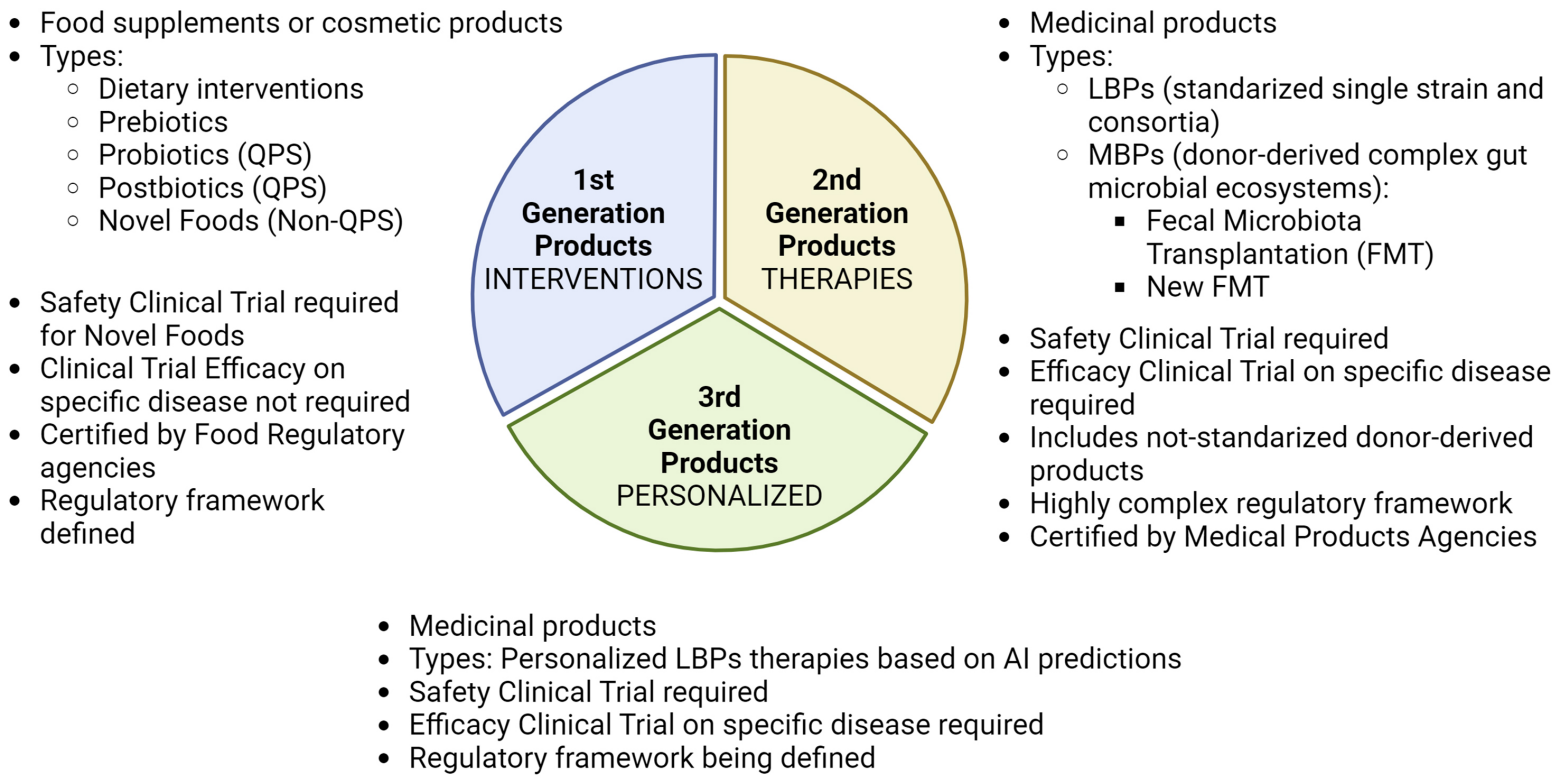
Schematic representation of the different strategies to modify the gut microbiota composition to obtain beneficial effects for human health and treatment for specific diseases.

A microbiome dietary intervention refers to dietary changes that aim to modulate the gut microbiota in order to improve human health, including but not limited to chronic diseases, such as inflammatory bowel disease and syndrome (IBD/IBS), Crohn’s disease (CD) and Type 2 diabetes^[72-74]^, and even to improve the effectiveness of cancer treatments such as immunotherapy and chemotherapy^[75,76]^ [Figure 1].

Several diets, including Mediterranean^[77]^, high fruits-vegetables-whole grains^[78]^, low-FODMAPs^[79]^, ketogenic^[80]^, and gluten-free diets, have been associated with specific gut microbiome compositions. Consequently, these diets have shown beneficial effects such as low inflammation, high insulin sensitivity, weight loss, improved cognitive function, and better immunological activity, among others. However, it is important to note that dietary interventions have to be chosen carefully based on each person’s baseline physiological state and gut microbiome composition and functional profile to exert the desired effect^[73,75,80-82]^.

Although many studies have shown that dietary interventions can specifically modulate gut microbiome composition, it remains unclear whether there are universal baseline features associated with microbiome responses to these interventions^[83]^. Future studies should identify and stratify subjects with a diet-responsive gut microbiota within study cohorts to maximize the success of these interventions^[84]^. It is also important to control for recent dietary patterns in observational studies, as interindividual differences in microbiota response to dietary interventions are known to exist^[85]^.

### Prebiotics

The most accepted definition of prebiotics describes them as substrates that are selectively utilized by host microorganisms, thereby conferring health benefits. The concept includes a substance, a physiologically beneficial effect, and a microbiota-mediated mechanism^[86]^ [Figure 1]. Prebiotics could be present in naturally or synthesized forms, including inulin, oligosaccharides, lactulose, pyrodextrins, dietary fibers, and resistant starches, among other candidates^[87]^.

Prebiotics play an important role in human health and are naturally included in low concentrations in different dietary food products. Prebiotic fibers are a direct energy source for some members of the microbiota, which will metabolize them, generating byproducts that can be used by other microbial species. This phenomenon is known as the substrate cross-feeding effect, and it can deeply impact the composition of the gut microbial ecosystem. An important example of the cross-feeding effect is the butyrogenic effect, which consists of the modification of the environment by acids produced during fermentation of prebiotics^[88,89]^.

Overall, prebiotics have shown potential for the treatment of various diseases by modulating the gut microbiome. This holds true for IBD, where prebiotics can serve as a complementary therapy, aiding in enhancing the composition of the gut microbiome and reducing inflammation in the gut^[90]^. In the gut-brain axis, a study conducted at Rush University found that prebiotics from plant origin, consisting mainly of fibers (inulin, resistant starch, resistant maltodextrin, and rice bran), can help treat Parkinson’s disease by promoting the growth of beneficial bacteria in the gut. The SCFAs produced by prebiotic metabolism can help reduce inflammation in the brain and improve motor function^[91]^. Beneficial effects have also been described in the treatment and prevention of diabetes and obesity^[92]^.

### Probiotics

**Figure 2:**
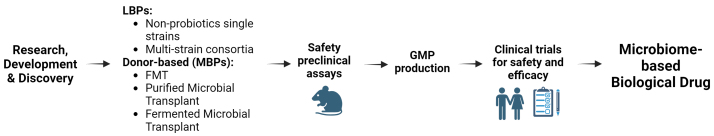
Schematic representation of steps required for approval of 2nd Generation microbiome-based therapies, also known as biological drugs. Both LBPs and MBPs therapies must be tested for safety in preclinical assays, produced under Good Manufacturing Practices, and tested in human clinical trials for safety and efficacy before being approved as biological drugs. LBPs: Live biotherapeutic products; MBPs: microbiota-based products.

In 2013, ISAPP defined probiotics as “live microorganisms that, when administered in adequate amounts, confer a health benefit to the host”^[93]^. Before commercialization, the specific microorganisms used as probiotics must be granted qualified presumption of safety (QPS) status. QPS status was developed by the European Food Safety Authority (EFSA)’s scientific panel as an approach to provide pre-evaluations on the safety of microorganisms intended for use in the food or feed chains^[94]^ [Figures 1 and 2].

The bacterial genera most used as probiotics are *Lactobacillus* and *Bifidobacterium*, bacteria found in the human gastrointestinal (GI) tract and in different dairy products. Other species approved by the EFSA belong to the genera *Streptococcus* and *Bacillus*, as well as the yeast *Saccharomyces*^[95]^. A list of QPS-recommended microorganisms for safety risk assessments carried out by EFSA is available and updated every 6 months at https://doi.org/10.5281/zenodo.1146566. Some fungi, bacteriophages, and bacterial taxa, such as *E. coli*, are currently excluded from the QPS assessments based on an ambiguous taxonomic position or the possession of potentially harmful traits by some strains of the taxonomic unit and require a specific assessment for which an application must be submitted^[94]^.

These bacteria are believed to play fundamental roles in human health status, contributing to the maintenance of gut homeostasis, supporting the function of the immune system, and protecting against certain pathogens^[15,95]^. Several in vitro and clinical trials have studied the general beneficial effects of different orally administered probiotics on host health, as well as their role in different diseases^[96]^.

### Synbiotics

The combination of probiotics and prebiotics is known as synbiotic products. Initially, synergistic synbiotics combined probiotics and prebiotics that enhance the functionality of such probiotics. However, most current symbiotic products are complementary synbiotics, which are defined by the ISAPP as “a mixture of live microorganisms and substrate(s) selectively utilized by host microorganisms that independently confer a health benefit on the host”^[97]^. Gomez Quintero *et al.* provide a review of recent examples of complementary and synergistic synbiotics, along with the rationale behind their formulation^[98]^.

### Postbiotics

A postbiotic, according to the definition proposed by Salminen *et al.*, refers to a “preparation of inanimate microorganisms and/or their components that confers a health benefit to the host”^[93]^. Postbiotics exert their benefits indirectly through modulation of the microbiome composition or directly by regulating the immune system^[99]^.

Most of the currently existing postbiotics are derived from bacteria of the *Lactobacillus* or *Bifidobacterium* bacterial genera, but also from yeasts such as *Saccharomyces cerevisiae*, mainly from the fermentation of these bacteria carried out in industrial processes^[100-105]^. Postbiotics are commonly metabolites or polysaccharides, substances with antioxidants, anti-inflammatory or even immunomodulatory properties^[15,95,100]^. One of these interesting metabolites is SCFAs, a product of bacterial fermentation of dietary fiber in the colon^[15]^. Thus, an important challenge in the development of postbiotics is the identification and isolation of the bioactive metabolites or proteins produced by these bacteria, usually done by chromatographic or metabolomic techniques^[100]^.

Novel postbiotics that could be approved as novel foods in regulatory terms if administered orally include whole bacterial cells that have been inactivated. For example, in 2019, pasteurized *Akkermansia muciniphila* was not approved as QPS but was for use as a novel food ingredient in the European Union. This species has been associated with a reduction in obesity and with an improvement in diabetes and various cardiovascular diseases^[106,107]^. In addition, oral administration of pasteurized *A. muciniphila* can improve obesity rates, reducing body weight^[108]^. A different study also showed that *A. muciniphila* can influence insulin resistance, improving glycemia values and glucose resistance^[109,110]^.

One of the main advantages of using postbiotics over probiotics is the reduced risk of bacterial translocation from the intestine to the systemic circulation and bacterial infection. Overall, they are considered safer, especially in neonates and vulnerable patients^[95,99,100]^. In addition, postbiotics have a longer shelf life and can be used more efficiently in topical formulations, as they do not have to be kept viable compared to probiotics. This is an advantage for their use in the cosmetic industry^[100]^.

In spite of certain health benefits reported for many probiotics, the benefit of these products is not always as robust as desired, most likely due to the high complexity of the gut microbiota ecosystem, and has been a controversial topic^[111-113]^. Among other issues, many probiotic strains fail to colonize the intestinal tract stably after ceasing the treatment and differences in efficacy depending on the strains of the bacteria used have been reported^[114]^. To overcome these limitations, great efforts have been invested in the development of more robust and efficacious therapies with proven effects and recognition as medical products, including live biotherapeutic products (LBPs) and FMT.

## 2ND GENERATION PRODUCTS - MICROBIOME-BASED THERAPIES A. LBPS

**Figure 3:**
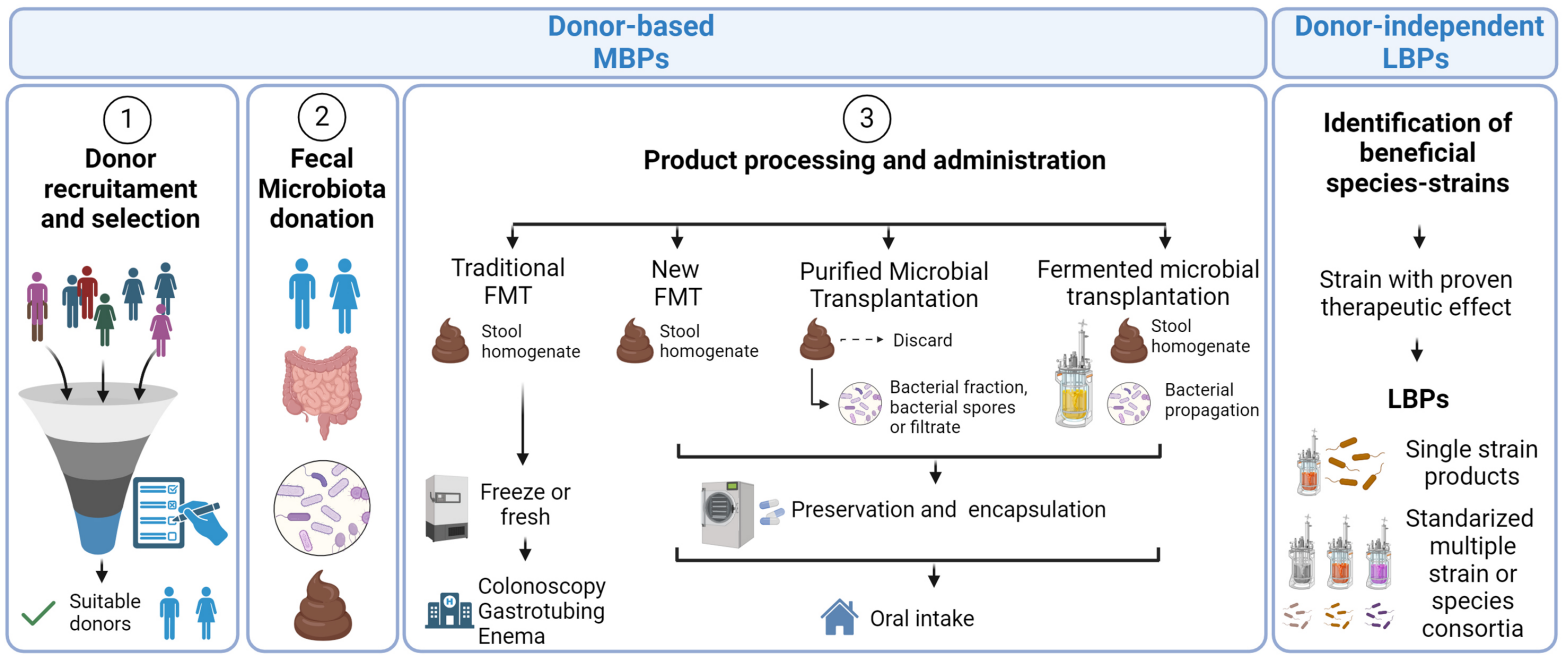
Schematic representation of 2nd Generation microbiome-based therapeutic approaches: Microbiome-based therapies can be divided into donor-based or donor-independent approaches. Donor-based approaches depend on the need for the donation of fecal material to produce the final therapeutic product. Donor-based approaches require the donation of fecal samples from individuals, which harbor a complex, non-standardized microbial community, and are subject to more intricate regulatory guidelines. On the other hand, LBPs are donor-independent microbiome-based products that consist of a single strain or standardized microbial consortia associated with the gut microbiome that have proven therapeutic effects in clinical trials. LBPs: Live biotherapeutic products.

Advances in sequencing technologies have resulted in the identification of new species important for human health and have led to the characterization of a wide range of genera and species that currently lack regulatory approval as probiotics. Among these are *Akkermansia muciniphila*, *Faecalibacterium prausnitzii*, *Veillonella, Ruminococcus*, *Christensenella minuta*, or *Bacteroides fragilis*^[106,107,115]^*.* These species, which were formerly referred to as Next Generation Probiotics, are currently defined as LBPs [Figures 1-3].

According to FDA, LBPs are defined as “a biological product that: (1) contains live organisms, such as bacteria; (2) is applicable to the prevention, treatment, or cure of a disease or condition of human beings; and (3) is not a vaccine”^[116,117]^. According to European Pharmacopeia, LBPs are considered “medicinal products containing live microorganisms such as bacteria or yeasts, which have a positive influence on the health and physiology of the host”, excluding fecal microbiota transplants and gene therapy agents^[118,119]^.

As medical products, LBPs must meet several conditions: (1) be effective, with expected benefits that outweigh its potential risks to patients; (2) be produced under good manufacturing practices (GMPs); (3) have identified and described the critical parameters that can influence batch reproducibility, product stability, product performance, and drug product quality^[106,107,115]^. Both probiotics and LBPs are live microorganisms, but the latter are included in medicinal products because they have been proven therapeutic or prophylactic activity in human diseases^[117]^ [Figure 2]. The development of LBPs is an active area of research, and the FDA is working to establish a regulatory framework for these products. The FDA-approved LBPs are a promising step towards the development of microbiome-based therapies. Because donor-dependent therapeutic products, such as whole or purified FMT, do not contain standardized composition and are excluded by European Pharmacopeia, we refer to such therapies as microbiota-based products (MBPs).

### FMT

The importance of a balanced gut microbial community has been largely established in recent years. A decrease in the diversity of this ecosystem, associated with certain health conditions or caused by different drug treatments, can have a negative impact on our well-being and even lead to infections^[69,120-125]^. Currently, one of the most effective treatments to reestablish the gut microbiome composition and diversity is to repopulate the GI tract with microbiota from a healthy donor^[12,126,127]^.

FMT involves the inoculation of a patient with the stool microbial community from a healthy individual [Figure 3]. The first reported case of FMT dates back to the fourth century^[128]^, but it was not until 1958 that this treatment gained popularity to control intestinal infections^[14,128,129]^. More than 70 clinical trials have proven the safety and the efficacy of FMT in treating patients with *Clostridium difficile* infection (CDI), which is the most common healthcare-associated infection, leading to substantial morbidity and mortality worldwide^[14,130-132]^. In this context, restoration of a naïve microbiota and keystone species (e.g., *Lachnospiraceae* and *Ruminococcaceae* members that are SCFA producers) can reestablish microbiome-induced colonization resistance by several mechanisms, including inhibition of germination and growth of *C. difficile* spores, competition for nutritional and colonization resources, and by reinstatement of the gut barrier and immune functions^[14,127,133,134]^.

### New FMT approaches

#### New formulations/strategies

The most common way of administering FMT treatment until now was through the introduction of homogenized fecal material (either from fresh or frozen samples) via colonoscopy, enema, or naso-oral gastric tubing^[135,136]^. Meta-analyses have shown that, among these, colonoscopy is the most effective mode of delivery, with no significant differences compared to oral administration (> 80% overall)^[132,137,138]^. Importantly, the upper delivery method has shown additional advantages in treating immune-related disease, likely due to immune regulation in the small intestine^[127,139]^.

Because FMT requires administration by medical staff, and the effectivity using enemas has been variable, public organizations and biotech companies are developing non-invasive, safe, and efficacious alternatives [Figure 3]. The use of oral capsules containing donor-derived lyophilized fecal material has proven to be almost as efficient as traditional FMT^[140]^. Moreover, to make more sanitary products and decrease the possibility of transferring undesired pathogens or toxic metabolites, companies are creating purified whole microbial ecosystem products.

Therapies not dependent on stool donations are being developed to overcome one of the major bottlenecks in the production of microbiota-based therapies - the availability of donated fecal material. Standardized microbiome-based products can range from multi-species consortia composed of microorganisms from different taxa, which provide general functions covered by the microbiota, to single-species products that are able to regulate very specific functions. We consider this type of standardized microbiome-based therapy as LBPs therapeutics (see discussion above), and currently, there are no LBPs (as standardized single or mix consortia) approved yet.

To support the development of complex microbiome-based therapies, the existence of stool banks is increasing worldwide^[141]^. Similar to how organ donations work, stool banks can coordinate donations from healthy individuals, perform thorough donor and sample screening to ensure product safety, store samples in adequate conditions, and provide the material to companies developing such products upon request. Moreover, banks can store individual samples on demand as a safety copy of their own microbiota, to have the option of receiving an autologous FMT treatment if desired in the future [Figure 1]. In spite of certain caveats, the usefulness of such organizations for the field of microbiome-based therapy is ever growing.

#### New disease targets

The great efficacy of FMT in treating CDI has spurred the investigation into its use to treat other health conditions (See Sorbara *et al.* for a detailed review)^[14]^. Because FMT success in CDI is driven, at least in part, by competition for nutritional resources, there are several CTs testing FMT potential to reduce or ablate colonization of the intestinal tract with multiresistant bacteria. So far, a reduction in recurrent infections of > 50% has been demonstrated^[131,142]^. In a similar way, modulating the native healthy vaginal microbiome is being used to improve women’s health.

Inflammatory bowel disease, which involves CD and ulcerative colitis (UC), is a GI disease characterized by chronic inflammation of the GI tract and changes in a shift in the microbiome composition^[143-145]^. UC is the second condition for which more CTs have been registered^[14]^. The efficacy compared to CDI is lower (35%-40% *vs.* > 70%), but positive results and the need for steroid-free therapies continue to move this research forward.

The treatment of Metabolic Syndrome with FMT has shown equivalent results, in which approximately only 50% of the recipients show clinical improvement. In-depth metagenomic analysis of several clinical trials has expanded our understanding of the ecological drivers of FMT at the strain level and has unraveled key determinants of success, such as engraftment or displacement of specific species and additional players such as viruses^[120,127,146,147]^.

Because the microbiome plays a key role in modulating the immune system, FMT treatment is being tested in an array of immune-related diseases, ranging from acute graft-versus-host disease (GVHD) to Type I diabetes or Multiple sclerosis, with variable but again promising results, especially in the treatment of GVHD^[14,139]^. Increasing data demonstrate that the ability of the gut microbiome to metabolize xenobiotic compounds and to regulate immune responses has profound consequences for anticancer therapies^[15,31,53]^. For instance, FMT can increase the effectiveness of immunotherapy and reduce the toxicity of chemotherapy, suggesting that microbiome-based treatments might become the new leading personalized anticancer therapies^[15,53,148]^.

#### Risks

The large amount of CTs performed to date have established that microbiota-based therapies are safe, but they are not void of some side effects and clinical risks that can be mitigated^[149]^. The most common side effects reported are mild diarrhea, abdominal cramping, and belching. These symptoms are generally resolved within 3 h and are also seen in control groups that receive their own fecal material. Administration through oral capsules has not shown a reduction of these side effects^[150]^.

A recent systematic review on the safety of FMT suggests that there can be additional risks including infectious (fever, bacteremia), autoimmune disease (peripheral neuropathy, Sjogren’s syndrome, idiopathic thrombocytopenic purpura, and rheumatoid arthritis, IBD flare among patients with UC), or metabolic syndrome^[12,149,151]^. However, these risks are seen in a minor percentage of cases and can be mitigated through strict screening of donors, which is becoming a mandatory procedure in clinical trials.

#### Regulatory framework

Until 2022, FMT and other microbiota-based therapies remained in a regulatory grey area in European countries. These therapies were not classified as drugs due to the wide diversity of gut microbiota composition between samples. Furthermore, because the microbiota is not an active human cell or a tissue, they were not considered within the scope of EU directive 2004/23^[152]^. In June 2022, the EU published a Faecal Microbiota Transplantation report, labeled EMA/204935/2022, with an updated regulatory framework in Europe, which should be used as a roadmap for the development of these products. Even though microbiota-based therapies are still not within a legislative framework at the EU level, the European Commission has published a report referring to them as biological medicinal products or biological drugs. Every Member State has decided to include these products in an appropriate regulatory framework at the national level, including quality and safety requirements. Most EU countries, such as Spain, France, or Germany, currently regulate MBPs as biological medicinal products, as suggested by the European Commission. Others, such as Belgium or Italy, presently regulate them within their national Tissues and Cells legislation^[153]^. Outside of the European Union, there is also a lack of harmonization at the regulatory level. The United Kingdom, Canada, Australia, and the United States are regulating MBPs as biological drugs with some exceptions^[154]^.

As biological drugs for human use, MBPs are subject to stringent regulations [Figure 2]. Mandatory regulations include the European Guideline on the requirements for quality documentation concerning biological investigational medicinal products on clinical trials, the ICH Harmonized Guideline for Good Clinical Practice and Regulation (EU) No 536/2014 of the European Parliament and of the Council on clinical trials on medicinal products for human use^[154]^. According to these regulations, the manufacturing process, control, and release of these products must be carried out under GMP standards and the clinical trials that guarantee the safety and functionality of the product under development must be performed^[154-156]^. Based on this, the first requirement for manufacturing is to have a facility certified as GMP by the corresponding regulatory agency and to have a license as a pharmaceutical manufacturing laboratory. It is also essential to register the dossier of the drug for approval at the regulatory agency, which exhaustively details all the drug information, including the name and general properties, traceability information, and safety tests of the raw materials used. Final approval of the product has not been granted until the first phases of clinical trials have been completed to corroborate its safety (Phase 1 toxicity trials) and efficacy for a given therapeutic indication (Phases 2 and 3).

Thanks to the efforts to harmonize the regulatory framework for MBPs, together with increasing investments and satisfactory results in clinical trials, there are currently three products with regulatory approval. These products are derived from healthy donors and focused on the treatment of *Clostridioides* infection and have been approved by the FDA or the Australian drug agency^[157-159]^. In spite of these efforts, the regulation of non-standardized therapies remains extremely complex across different countries, making the development of standardized and equally efficacious products essential as we move forward.

## 3RD GENERATION PRODUCTS TO MODULATE THE MICROBIOTA - PERSONALIZED MICROBIOME-BASED THERAPIES

Traditional medicine is largely based on statistical averages on responses to treatment - the treatment that works for most people is applied to everyone - generating mixed results. This approach does not consider that the unique human and microbial genetic profile of each patient impacts disease profile and response to treatment, and all should be considered when choosing therapeutic options. This is the basis for precision medicine - using an individual’s unique genetic signature together with microbiome profile, tumor genetics (in case of cancer treatment), and other patient variables - to empower health professionals to determine the best course of action for each individual^[66]^.

Shotgun metagenomics has enabled the generation of vast amounts of information on the human microbiome across different parts of the body and diverse health states^[9,160-163]^. Combined with traditional computational methods, such as Principal Coordinate Analysis, this technique has shed light on key taxonomical and functional microbial features associated with certain medical conditions, healthy states, and responses to therapeutic drugs. However, these methods are costly, ineffective for large-scale analyses and require certain information and selection of study variables. These limitations have sped the use of data mining and enhanced artificial intelligence (AI) approaches, such as advanced deep learning (DL) algorithms to discover key signatures and complex host-microbiome interactions [Figure 3].

The information obtained from microbiome studies in clinical trials is extensive and heterogeneous, typically consisting of microbiome, clinical, and environmental data. AI algorithms integrate these pieces, revealing hidden patterns and relationships that would otherwise remain elusive. Through AI - machines that mimic cognitive functions associated with the human mind, such as learning and problem-solving - a computer system employs mathematical and logical techniques to learn from available information and make decisions^[164,165]^. Machine learning (ML) is a method of implementing AI wherein algorithms are trained with data to learn and grow. ML systems extract and transform selected features from raw data into a learning subsystem that can use them to detect or classify patterns in the input. To facilitate this, more sophisticated forms of ML that do not need preselected features were developed and are known as DL^[166]^. Overall, DL is a subset of ML and ML is a subset of AI^[167]^.

ML models trained on microbiome data from healthy and diseased individuals can identify patterns associated with each group and discover potential biomarkers for early disease detection and monitoring^[168-171]^. Currently, a few ML algorithms have been used in clinical studies^[172]^. To highlight two of the most recent ones, Su *et al.* designed a fecal microbiome-based ML multi-class model for disease diagnosis that achieved high performance in classifying patients with or without colorectal adenomas, CD, colorectal cancer, cardiovascular disease, diarrhea-dominant irritable bowel syndrome, post-acute COVID-19 syndrome, and UC^[170]^. On their part, Radjabzadeh *et al.* found an association between 13 bacterial taxa (including genera *Eggerthella*, *Subdoligranulum*, *Coprococcus*, *Sellimonas*, *Lachnoclostridium*, *Hungatella*, *Ruminococcaceae*, *Lachnospiraceae*, *Eubacterium ventriosum* and *Ruminococcusgauvreauiigroup*, and family *Ruminococcaceae*) of the gut microbiota and symptoms of depression^[173]^. Moreover, combining multiomics data (genomics, transcriptomics, and metabolomics) with clinical and demographic information, ML models have already been used to uncover complex interactions between microbiome and human host^[174]^.

A complete integration of the microbiome composition (taxonomy), microbiome metabolic capacity, microbiome-host metabolic interactions, host genome, and medical history, obtained from ML and DL algorithms, will allow for the identification of key therapeutic targets and will provide critical knowledge to develop novel interventions and create minimal and even personalized consortia for different patients and disease conditions^[175]^. In the same way, AI analysis of microbiome data alongside clinical information and treatment responses will identify patient subgroups that are more likely to benefit from specific interventions, including those in which the microbiome is involved. Overall, AI opens the door to the diagnosis of diseases based on microbiome profile analysis, and to the development of personalized treatments to reestablish a healthy microbial profile. Ultimately, the use of AI to enhance microbiome-based therapeutics will improve patient outcomes and decrease risks and production limitations associated with 2nd generation microbiome therapies.

## FUTURE PERSPECTIVES AND CONCLUSIONS

As we commented previously, the successful story of FMT as a treatment for *C. difficile* has opened the door to research and regulation of microbiome-based therapies. With the approval of three therapies by the pertinent regulatory bodies and the increasing number of clinical trials worldwide, it is evident that microbiome-based therapies are here to stay. Nevertheless, significant efforts still need to be made to completely understand the mechanisms of action by which these therapies exert their effect, as well as to develop new formulations aimed at improving their scalability and reinforcing their safety. In addition, the close relationship between gut microbiome and immune system modulation, altogether with the development of new bioinformatic tools, such as AI, could lead to the blooming of new patient-tailored solutions.

Microbiome research is a rapidly evolving field, demanding full recognition not only from the regulatory agencies, but also from researchers and industrial partners. This collective acknowledgment is crucial for devising optimal solutions to various health issues and technological challenges where the gut microbiota could be implicated.

## DECLARATIONS

### Copyright

© The Author(s) 2024.
